# Saving time and money: a validation of the self ratings on the prospective NIMH life-chart method (NIMH-LCM)

**DOI:** 10.1186/1471-244X-14-130

**Published:** 2014-05-07

**Authors:** Christoph Born, Benedikt L Amann, Heinz Grunze, Robert M Post, Lars Schärer

**Affiliations:** 1 Department of Psychiatry, Ludwig Maximilians-University, Nußbaumstr. 7, Munich 80336, Germany; 2 FIDMAG Research Foundation Germanes Hospitalàries, Barcelona, Spain; 3 CIBERSAM, Barcelona, Spain; 4 Institute of Neuroscience, Newcastle University, Newcastle, UK; 5 Bipolar Collaborative Network, Bethesda, MD, USA; 6 Department of Psychiatry, Albert Ludwig-University, Freiburg, Germany

**Keywords:** Bipolar disorders, Mania, Depression, Long-term course, Life-chart

## Abstract

**Background:**

Careful observation of the longitudinal course of bipolar disorders is pivotal to finding optimal treatments and improving outcome. A useful tool is the daily prospective Life-Chart Method, developed by the National Institute of Mental Health. However, it remains unclear whether the patient version is as valid as the clinician version.

**Methods:**

We compared the patient-rated version of the Lifechart (LC-self) with the Young-Mania-Rating Scale (YMRS), Inventory of Depressive Symptoms–Clinician version (IDS-C), and Clinical Global Impression–Bipolar version (CGI-BP) in 108 bipolar I and II patients who participated in the Naturalistic Follow-up Study (NFS) of the German centres of the Bipolar Collaborative Network (BCN; formerly Stanley Foundation Bipolar Network). For statistical evaluation, levels of severity of mood states on the Lifechart were transformed numerically and comparison with affective scales was performed using chi-square and t tests. For testing correlations Pearson´s coefficient was calculated.

**Results:**

Ratings for depression of LC-self and total scores of IDS-C were found to be highly correlated (Pearson coefficient r = −.718; p < .001), whilst the correlation of ratings for mania with YMRS compared to LC-self were slightly less robust (Pearson coefficient r = .491; p = .001). These results were confirmed by good correlations between the CGI-BP IA (mania), IB (depression) and IC (overall mood state) and the LC-self ratings (Pearson coefficient r = .488, r = .721 and r = .65, respectively; all p < .001).

**Conclusions:**

The LC-self shows a significant correlation and good concordance with standard cross sectional affective rating scales, suggesting that the LC-self is a valid and time and money saving alternative to the clinician-rated version which should be incorporated in future clinical research in bipolar disorder. Generalizability of the results is limited by the selection of highly motivated patients in specialized bipolar centres and by the open design of the study.

## Background

Bipolar disorder is a life long and complex clinical entity. Various manifestations of depression, mania, hypomania, and mixed states characterize the mood-fluctuations, with depressive episodes prevailing over manic or hypomanic episodes [1,2]. Even though research on bipolar disorder has increased in the last two decades, accurate diagnosis is still often delayed by 8 to 10 years [3,4]. Furthermore, finding optimal treatment gets more complex as more options are available and as the course varies markedly within the bipolar spectrum, including rapid-cycling forms, mixed or psychotic features. The illness also often gets complicated by psychiatric comorbidities, such as substance use disorders, anxiety or personality disorders. A further risk factor for a poorer outcome might be the diagnosis bipolar II disorder with more and longer duration of episodes and shorter duration of well intervals than in bipolar I patients [5].

Therefore, the accurate assessment and documentation of the long-term course is pivotal to optimizing treatment and improving the outcome of each individual bipolar patient [6]. One of the most widely used approaches to document the course of bipolar disorders is based on the observational research of Kraepelin in the early years of the 20th century. The US National Institute of Mental Health modified the methods of Kraepelin and created the Life-chart Methodology (NIMH-LCM). The major variable in the NIMH-LCM is four levels of depression based on the degree of functional impairment related to affective symptoms (from −1 slight or mild to −4 severe and incapacitating depression) and similarly four degrees of mania (from +1 mild to +4 severe mania), with 0 representing a balanced, well-functioning euthymic state. Additionally, medications, life events, drug abuse, hours of sleep, irritability, ultradian cycling, and co-morbidities are documented on a daily basis in the prospective Life-chart.

By evaluating these items on a daily basis, decreasing or increasing frequency and severity of episodes over time, responders and non-responders to different medications and the impact of drug abuse and life events can be easily identified. Also assessment of long term patterns of response and sometimes its waning, as the phenomena of tolerance and discontinuation refractoriness, can be readily identified. Increasing appreciation of the long term course of illness, and enhancement of the therapeutic alliance and compliance are also by-products of the LCM. We previously demonstrated that patients using the NIMH-LCM regularly experience an increase in euthymic days and a decrease in (subsyndromal) depressive and (hypo)manic days [7].

The NIMH-LCM contains a retrospective chart using monthly ratings [8] and a prospective chart using daily ratings [9]. Furthermore, there is a patient-rated version of the Life-chart (LC-self) and a clinician-rated version (LC-clinician) for both time domains. The prospective LC-clinician is predominantly used in clinical and scientific settings and has shown good validity and reliability [10,11]. The LC-self collects the same information and could represent a rapid and time saving source of information for the clinician about the course of illness since the last visit of the patient [12]. However, only the LC-clinician has been validated so far.

Although the LC-self needs to be more formally validated for use in future trials, we performed this first analysis to test whether the prospective LC-self shows similar properties to the LC-clinician by comparison to various mood scales in participants of the Naturalistic follow-up study (NFS) of the Bipolar Collaborative Network (BCN; formerly Stanley Foundation Bipolar Network) [13].

## Methods

We used the same methodology as proposed in earlier validation studies of the clinician-LC [10,11]. The study was approved by the local ethical committees in Munich and Freiburg (reference number Munich 112a/99 and Freiburg 114/99). After signing informed consent, patients from both German sites of the international BCN, Munich and Freiburg, were included into the NFS. Inclusion criteria for entry into the NFS were the diagnosis of bipolar I, bipolar II, bipolar not otherwise specified or schizoaffective disorder, bipolar subtype, and being willed to attend at least monthly visits, participate in detailed evaluations and prospective lifecharting. Patients were only excluded in the case of current active substance abuse or imminent suicidal threat.

The detailed procedure of the NFS protocol has been reported elsewhere [9,14]. The NFS was an open trial to gather data about the naturalistic course of illness and primarily not designed for the validation of the LC-self. Nevertheless, we took advantage of this data to validate also the LC-self. In line with the protocol of the NFS every subject provided his prospective LC-self at each monthly visit. The NFS protocol also required several other cross sectional scales used at each visit, amongst them the Young Mania Rating Scale (YMRS; [15]), the Inventory of Depressive Symptoms-Clinician Version (IDS-C; [16]), and the Clinical Global Impression-Bipolar Version (CGI-BP; [17]). Unlike the IDS-C and the YMRS, the CGI-BP ratings display a single estimation of the severity of the overall mood syndrome (0 to 7) and not evaluating different mood symptoms. Severity of the acute manic syndrome is assessed by the CGI-BP IA ratings, severity of the acute depressive syndrome by the CGI-BP IB rating, and total severity of the illness by the CGI-BP IC rating [17]. The rating of the YMRS includes the last two days, the IDS-C the last week and the CGI-BP the whole time since last visit, including the maximum or worst rating since last visit, while the Lifechart was designed for daily rating. For validation with the cross sectional scales, we used the average Lifechart rating of the same time frames used for each scale as noted above. Raters were not blinded to the results of the cross sectional scales when they were rating the LC-clinician.

All clinicians of the BCN received specific training for using these instruments and inter-rater reliability was checked. Patients also received standardized instructions how to perform lifecharting, suggesting that it should be carried out at the end of each day and linked to some routine bed time activity such as brushing teeth or taking medications to facilitate the habit and reliability of completing the LC self. For data processing the graphic presentation of the Life-chart was then transformed into numeric digits. As stated above, every level of mood reaches from severe depression to severe mania being represented by a number (from −4 to +4), while 0 was considered as an euthymic state. Each severity grade of illness was primarily linked to the degree of functional incapacitation in a patient´s usual social, educational, or occupational role and not necessarily with the subjective perception of mood in order to facilitate the recall over both retrospective and prospective time domains. Severe depression (−4) was associated with essentially complete incapacitation, high moderate depression (−3) with great extra effort required to complete needed tasks, low moderate depression (−2) with some extra effort being required, and mild depression (−1) with awareness of depression, but associated with little or no functional incapacity. Euthymia (0) was defined as functioning without any restrictions in daily life. Mild hypomania (+1) imply even more productivity than usual, low moderate hypomania (+2) some difficulty in goal directed activity, high moderate mania (+3) with great difficulty in goal directed activity, and severe mania (+4) essentially incapacitaty or hospitalization.

### Statistics

Data were stored on an SQL-Server (DB2, Version 8.2). For extraction of data the databank-management-system OMNIS-Studio (Version 4.1) was used. Statistical calculation was performed by using the SPSS software (Version 11). For comparison of demographic data the chi-square test and the t test were used, and to estimate correlations Pearson´s coefficient was calculated.

## Results

Evaluable data sets of 108 of 140 patients participating in the NFS from 1999 to 2002 at the German sites of the BCN were available for the comparison of the LC-self with the IDS-C, YMRS and CGI-BP. Basic demographic data of our sample are given in Table 1. In this particular subset of patients only 2 patients fulfilled diagnostic criteria of schizoaffective disorder, bipolar type.

**Table 1:** Demographics

	**Female (n)**	**Male (n)**	**Total (n)**
Bipolar I (n)	38 (74,5%)	40 (70,2%)	78 (72,2%)
Bipolar II (n)	12 (23,5%)	16 (28,1%)	28 (25,9%)
Schizoaffective, bipolar subtype (n)	1 (2%)	1 (1,8%)	2 (1,9%)
Age (mean ± SD)	38,8 ± 14,3 years	39,8 ± 12,4 years	39,3 ± 13,3 years
total (n)	51 (47,2%)	57 (52,6%)	108 (100%)

**Figure 1:**
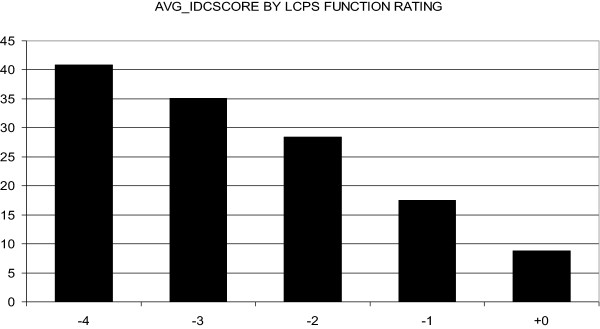
Average IDS-Clinician Ratings for the lifechart functing rating values in the depressive and euthymic range (severe depression: −4; high moderate depression: −3; low moderate depression: −2; mild depression: −; euthymia: +0).

As a first step, we compared the daily LC-self versus the IDS-C rated depressive symptoms. A strong correlation was found between ratings of depression severity on the LC-self and the total scores of the IDS-C (Pearson coefficient r = −.718; p < .001). We found a linearity of this correlation when charting the average values of the overall severity of depressive symptoms measured by the IDS-C scores and the different levels of functioning of the Life-chart (Figure 1).

**Figure 2:**
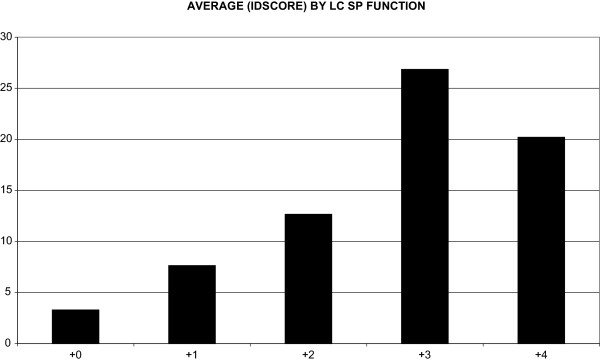
Average YMRS clinician ratings for the lifechart functioning rating values in the manic and euthymic range (severe mania: +4; high moderate mania: +3; low moderate mania: +2; mild mania: +1; euthymia: +0).

As second step, we also compared the mania ratings on the LC-self with the YMRS scores. Again, results were statistically significant (Pearson coefficient r = .491; p < .001) and following a linear model (Figure 2).

Furthermore, we found a good correlation for the CGI-BP IA with the LC-self for mania (Pearson coefficient r = .488; p < .001), for the CGI-BP IB with the LC-self for depression (Pearson coefficient r = .721; p < .001), and for the CGI-BP IC ratings which were correlated with the absolute maximum of the LC-self rating (Pearson coefficient r = .65; p < .001).

## Discussion

As stated in the introduction, the NIMH-LCM has been found to be a useful tool both in clinical practice and trials for the assessment and documentation of the long-term course of bipolar disorders [10]. It has been widely used to evaluate therapeutic interventions [18,19], especially in studies of the SFBN. While the LC-clinician has shown good validity and reliability when tested against standard scales [10,11] such information had not been previously available for the LC-self. To our best knowledge, this is the first analysis aimed at a preliminary validation of the daily prospective LC-self against well-validated clinician-rated cross-sectional psychometric scales for the assessment of manic and depressive symptoms. In a reasonable number of bipolar outpatients, we found a highly significant correlation and good concordance between the daily LC-self and the clinician-rated scales for mania (YMRS and CGI-BP IA), depression (IDS-C and CGI-BP IB) and for the overall mood state (CGI-BP IC), suggesting good reliability and validity for the LC-self. Results are very similar to those of the earlier validation studies of the LC-clinician [10,11], the methods of which we replicated. While the NFS was not performed prospective for the primary purpose of validating the LC-self, we nevertheless used this opportunity to retrospectively examine the data for this preliminary first look at its performance characteristic and analysis in comparison to well-known clinician ratings.

Despite the general robust correlation demonstrated, the results need to be viewed with caution and the generalizability of our findings may be limited. Interestingly, correlation of the LC-self on severe mania (level +4) was less consistent than for moderate mania (level +3) as shown in Figure 2. As might be expected there seems to be a less good correspondence of the LC-self on severe mania (level +4) than for moderate mania (level +3), as shown in Figure 2. There are several possible interpretations, such as the lack of insight patients often present with when severely manic. Furthermore, the lack of an adequate sample size and therefore insufficient data at this severity level might be also a likely contributor because patients in this study were outpatients and rarely showed full-blown manic symptoms.

In addition, the patient cohort in this trial was highly selective as subjects were motivated to participate in a longitudinal clinical research effort, agreed to be carefully studied and rated, and they received detailed instruction sessions on the use of the daily Life-chart. For a reliable future use of computer-based self-ratings for clinical and scientific purposes, the provision of comprehensive instructions to patients, e.g. with training videos or other methods, might be essential. Moreover, means of monitoring consistency in completion of the LC-self ratings and/or encouraging their completion by phone calls, automated text messages, or other modes of feedback might also be desirable.

Another methodological limitation is that NFS clinicians were at the same time raters and treating physicians, and thus were not blinded to the LC-self when rating the YMRS, IDS-C and CGI-BP which increases risk of bias. Finally, missing data points, as seen in most other long-term studies, did also occur in the NFS program which may be problematic for such trials in general [20]. This problem might improve with the establishment computer-based self-ratings, as this would facilitate data-management, the generation of automatic reminders, and as well as reduce time- and money-consuming procedures associated with paper and pencil forms.

In the past years several approaches have been used to optimize the long-term documentation of the course in bipolar disorders, but few have utilized daily ratings. Small time windows, however, are of critical importance given the extreme diversity of episode duration, frequency, and patterning inherent in bipolar disorder [2,5,21,22] and its multiplicity of Axis I, Axis II, and medical co-morbidities.

Other methodological approaches to clinician-versus patient-rating and usage of paper sheets versus computer-based programs for data-collection have been utilized.

The NIMH-LCM contains both self- and clinician-ratings, and the studies of the SFBN used paper sheets for data collection. The STEP-BD Blank Mood Chart, which was developed by Sachs and coworkers (available at http://hppt://www.manicdepressive.org/images/samplechart.gif) is a self-rating instrument providing data also on paper sheets. The Internal State Scale is another self-rating instrument which showed good reliability and validity in the early 1990s [23]. Further options include the Patient Mood Chart (PMC), validated in a small cohort in a nine month trial [24], and the ChronoRecord, a personal-computer based program [25], which has been validated for depression and mania [26,27]. Another electronic diary was developed by one of the authors (L.S.) for the use on a palm-top computer. It uses the NIMH-LCM for documentation and has shown good results in a feasibility study [12] . Today the use of this program, named “Personal Life-chart” (http://www.bipolar.de/) is no longer restricted to palm-tops.

The implications of having an easy-to-use partially validated self rating instrument available for routine clinical use in outpatient treatment are wide ranging. Self-rating is essential to complement the information of the clinician rating, and helps clinicians to gain insight into patients self-perceptions and details of illness variation, necessary for the evaluation of short and long term treatment response and remission [28]. Daily self-ratings can be easily and rapidly performed by the patient, and the patient plays an active part in the evaluation of the treatment process in terms of responsibility, self-monitoring, and seeking early treatment for emergent symptoms. In appropriately motivated populations the LC self, also would have a great potential for its use in a research setting.

The LCM and some of the other above mentioned methods for documentation of the long-term course offer obvious advantages in relation to other cross sectional psychometric scales. First, the daily ratings should be highlighted as necessary for the identification and documentation of frequent mood swings which occur in a much higher proportion of patients than previously recognized. For instance, ultra-rapid cycling with four or more episodes/month occurs in some 40% of patients and ultradian cycling in some 19%. It has also been shown that the onset of depressive episodes is faster in patients with bipolar versus unipolar depressive disorder [29]. Capturing depressive (or manic) symptoms early will enhance the chance of timely and successful intervention.

Another advantage of the LCM might be the fact that the degree of mood-driven functional impairment is the rated measure of severity rather than the rating of individual mood symptoms. Remembering whether one was able to go to work or function with no, some or much difficulty is much more easily judged and remembered across weeks to months than subtle differences in specific mood symptoms. This also may make this type of self-ratings more reliable as the degree of functional incapacity is a behaviour manifestation and might be less affected by subjectivity than evaluation of a multiplicity of internal mood and anxiety symptoms. However, self-rating of mood-driven functional impairment is also subject to bias because of the subjective view of the amount of difficulty involved in completing ones’ educational, occupational, or social roles. The most critical items in terms of diverging opinion between clinicians and patients were in the severe range of manic symptoms which may be confounded by poor insight. It is widely recognized that the patients’ estimation of manic symptoms is a more difficult issue than self-rating of depression (e.g., [30]).

A further justifiable criticism of the NIMH-LCM is that severity of mood and functional impairment can sometimes be markedly dissociated. For these instances the NIMH-LCM also has another rating of mood on a 0 (most depressed ever) to 100 (most activated or manic ever) scale (where 50 is balanced) that can be used to discern such disjunctions between mood and functional incapacity.

Another issue is that many scales have been developed for rating unipolar depression and do not capture the complexities of affective symptoms in bipolar disorder. A helpful rating scale for depression in bipolar disorders has been developed by Berk et al. which also captures mixed symptoms [31]. Self-rating scales for mania are scarce and seldom used in clinical trials [30] but the AS-18, a self-rating scale for bipolar disorder, has been developed to assess depressive, manic and mixed affective states [32]. This scale is claimed to be time-efficient and an alternative to the use of two different observer rating scales for mania and depression.

## Conclusions

In conclusion, we found good correlations of standard affective rating scales with the LC-self. The use of the LC-self might facilitate detailed data-collection in future short- and long-term studies and reduce time- and money-consuming hetero-rating procedures. Furthermore, it may even be amenable for large practical clinical trials in the community, which are indispensable for the study of the relative effectiveness and optimization of our current treatment option for this complex and dynamic disease.

## Competing interests

The authors declare that they have no competing interests.

## Authors’ contributions

CB was involved in interpretation of data and drafted the paper. BLA was involved in recruitment of patients and proof reading. HG worked as principal investigator for the study and was involved in proof reading. RMP was involved in scale devolepment, study organization, and editing the manuscript. LS was involved in recruitment of patients, analyzing the data and proof reading. All the authors have revised the manuscript for important intellectual content, and have read and approved the final manuscript.

## Pre-publication history

The pre-publication history for this paper can be accessed here:

http://www.biomedcentral.com/1471-244X/14/130/prepub
